# Clinical Characteristics and Lung Function in Older Children Vertically Infected With Human Immunodeficiency Virus in Malawi

**DOI:** 10.1093/jpids/piv045

**Published:** 2015-08-25

**Authors:** Thandie Mwalukomo, Sarah J. Rylance, Emily L. Webb, Suzanne Anderson, Bernadette O'Hare, Joep J. van Oosterhout, Rashida A. Ferrand, Elizabeth L. Corbett, Jamie Rylance

**Affiliations:** 1 Malawi-Liverpool-Wellcome Trust Clinical Research Programme, Blantyre; 2 Liverpool School of Tropical Medicine, United Kingdom; 3 College of Medicine, University of Malawi; 4 London School of Hygiene & Tropical Medicine, United Kingdom; 5 Dignitas International, Zomba, Malawi; 6 Medical Research Council Unit, Gambia; 7 University of St Andrews, United Kingdom

**Keywords:** case definition, chronic lung disease, HIV, infectious disease transmission, respiratory function tests, vertical

## Abstract

**Background:**

Antiretroviral therapy (ART) has led to increased survival of children with vertically acquired human immunodeficiency virus infection. Significant morbidity arises from respiratory symptoms, but aetiology and pulmonary function abnormalities have not been systematically studied.

**Methods:**

Human immunodeficiency virus-positive children aged 8–16 years were systematically recruited within clinics in Blantyre, Malawi. Clinical review, quality of life assessment, spirometry, and chest radiography were performed.

**Results:**

One hundred sixty participants had a mean of age 11.1 (range, 8–16) years and 50.0% were female. Cough was present in 60 (37.5%) participants, and 55 (34.4%) had moderate or severe dyspnoea. Thirty-four (22.1%) participants had digital clubbing. Thirty-three (20.6%) participants were hypoxic at rest. One hundred eighteen (73.8%) of the children were receiving ART; median CD4 count was 698 cells/µL in these compared with 406 cells/µL in ART-naive individuals (*P* < .001). From 145 spirometry traces (90.6%), mean forced expiratory volume in 1 second (FEV_1_) and forced vital capacity **(**FVC) were 1.06 and 0.89 standard deviations below predicted mean, respectively. Twenty-one (14.5%) traces demonstrated obstructive defects and 26 (17.9%) reduced FVC. Lung function abnormality was not associated with any clinical findings. Of the 51 individuals with abnormal lung function, the mean increase in FEV_1_ after salbutamol was 3.8% (95% confidence interval, 0.02–7.53). “Tramlines” and ring shadows were seen on chest radiographs in over half of cases.

**Conclusions:**

Symptoms of chronic lung disease were highly prevalent with 2 main clinical phenotypes: “cough” and “hypoxia”. Lung function abnormalities are common, poorly responsive to bronchodilators, and apparent throughout the age range of our cohort. Pathological causes remain to be elucidated. Cough and hypoxic phenotypes could be a useful part of diagnostic algorithms if further validated.

An estimated 3.2 million children are living with human immunodeficiency virus (HIV) [1], most of whom live in sub-Saharan Africa. Approximately 199,000 children were newly infected in 2013. Scale-up of antiretroviral therapy (ART) provision is likely to contribute to improvements in survival amongst children with HIV [2]. However, in sub-Saharan Africa, only 22% of HIV-positive children are receiving ART, lagging behind 39% coverage in adults [1]. Prevention of vertical infection through Prevention of Mother-to-Child Transmission (PMTCT) program is effective, but worldwide coverage requiring ART has been suboptimal. An estimated 1%**–**3% of all 10-year-olds in Southern Africa are HIV-infected, long-term survivors, with 68% of eligible women receiving ART as PMTCT in 2013 compared with 33% 5 years previously [1]. In a hospital-based study from Zimbabwe, advanced HIV infection was the single most common cause of admission and death in adolescents [3].

With longer survival, lung effects of HIV become more prominent. Human immunodeficiency virus-infected children in sub-Saharan Africa are subject to frequent pulmonary infections [4], and they commonly develop chronic cough in older childhood. In a Zimbabwean study of 116 HIV-infected adolescents receiving HIV care, dyspnoea was often disabling, and resting hypoxia or desaturation at submaximal exercise was present in 40% of patients [5]. Chest radiographs were abnormal in two thirds of patients, characterized by ring and tramline opacities, whose presence is unrelated to clinical symptoms. High-resolution computerized tomography (HRCT) scans suggested small airways disease as the most common cause of infection; however, bronchodilator response was not assessed [6]. Despite this, data on symptoms of chronic lung disease (CLD) and lung function testing, particularly in developing countries, are lacking.

We describe the burden and clinically useful phenotypes of CLDs, and we assess the bronchodilator response with inhaled beta-agonist therapy in HIV-infected children aged 8 to 16 receiving HIV care in Blantyre, Malawi.

## METHODS

### Participants

Participants were recruited from outpatient HIV clinics in Queen Elizabeth Central Hospital, Blantyre between July and December 2011. The first 3 eligible patients per day were included. Patients were not eligible for recruitment if they (1) resided outside urban Blantyre, (2) were currently taking tuberculosis (TB) treatment, (3) had Kaposi's sarcoma, (4) reported acute respiratory symptoms (≤1 week of any one or more of fever, purulent sputum, pleuritic chest pain), or (5) required emergent hospitalization.

At baseline, we assessed medical history, symptoms, quality of life, and functional status by standardized verbal questionnaires administered in the local language. Examination included assessment of finger clubbing, growth, and World Health Organization (WHO) clinical staging of HIV disease. CD4 count was performed, and TB smear and culture were done in all participants who could spontaneously expectorate. Participants performed a 200 meter submaximal walk test, unless contraindicated due to resting hypoxia (SpO_2_ <92%) or tachypnoea (>24/minutes). Within 2 weeks, participants had spirometry unless there was evidence of TB or acute respiratory illness [7]. Chest radiographs were reported by 2 independent clinicians using a standardized scoring system [6], with discrepancies resolved by consensus.

### Quality of Life Assessment

In the absence of a disease-specific tool, quality of life was assessed using the Cystic Fibrosis Questionnaire-Revised ([CFQ-R] for 6- to 13-year-olds and their carers in parallel). These incorporate 9 quality of life domains (physical, school, vitality, emotion, social, body image, eating, treatment burden, health perception) and 3 symptom domains (respiratory, digestion, weight). Translation was done by 2 independent translators, collation by consensus, and back-translation for accuracy [8].

### Spirometry and Radiology

Spirometry was performed according to ATS/ERS guidelines [9] by experienced nursing staff and a respiratory physician. Forced exhalation following maximal inspiration was recorded while seated using an EasyOne World spirometer (ndd, Zurich, Switzerland). Up to 8 trials were recorded, and these were assessed by 2 clinicians independently for quality. The best forced expiratory volume in 1 second (FEV_1_) and forced vital capacity (FVC) values from 3 admissible traces were included for analysis. Primary reference values were taken from the Global Lung Initiative [10], using the 5th centile of the reference population as the lower limit of normal ([LLN] 1.64 standard deviation [SD] below the mean). Results of FEV_1_, FVC, FEV_1_:FVC, and forced expiratory flow at 25%–75% are reported as residual SDs (z-scores). Participants with FEV_1_ and FEV_1_:FVC ratio of less than the LLN were classified as having obstructive spirometry. Where FVC was reduced below LLN and FEV_1_:FVC was not, we recorded “reduced FVC” because we were unable to measure total lung capacities. In participants with abnormal spirometry, testing was repeated after nebulized salbutamol (2.5 mg via face mask). Reversibility was defined as improvement of ≥12% in best FEV_1_ or FVC [9]. Participants meeting this criterion were prescribed inhaled salbutamol via metered dose inhaler with an Aerochamber device (GSK, UK), 200 µg at least twice a day, and additionally when symptomatic. These participants returned for clinical reassessment after 4 weeks of treatment.

Secondary interpretation used a locally derived reference range [11], with LLN of 80% predicted. Results are presented as “percentage of normal” given uncertain population standard errors.

### Laboratory Methods

CD4 counts were determined by flow cytometry (BD FACSCount, Franklin Lakes, NJ). Participants with cough were asked to provide 2 sputum specimens. Concentrated decontaminated sputum specimens were examined with auramine and cultured using Mycobacterial Growth Indicator Tubes (MGIT; BD, Franklin Lakes, NJ). Positive mycobacterial cultures were confirmed by Ziehl-Neelsen staining and speciated using the Hain assay (Hain LifeScience GmbH, Germany).

### Ethical Approval

Ethical approval was obtained from the College of Medicine Research Ethics Committee, Malawi (P.02/11/1039) and the London School of Hygiene and Tropical Medicine Ethics Committee (approval #5964). Informed written or witnessed thumbprint participant assent and parental or guardian consent were required for recruitment.

### Analysis

Univariable associations of abnormal lung function were assessed using logistic regression, with predictors at *P* < .10 taken forward to a multivariable model in addition to age and sex.

Exploratory analysis was used to compare different prototype definitions of CLD, including 2-way associations between individual variables, aiming for a definition that could be applied in outpatient clinics where investigations are limited to oximetry and symptom screening. We assessed univariable relationships, looking for a clinically useful phenotype. Multivariable logistic regression was then used to identify independent variables independently associated with these phenotypes. A hierarchical approach to modeling (2 levels: distal and proximal) was used to account for factors that affect the lungs indirectly and directly, respectively [12]. Distal (indirect) factors were as follows: stunting, orphanhood, and variables selected a priori for inclusion including sex, age, ART, and CD4 count. Proximal (direct) factors included symptoms, physiological observations, and radiographic and spirometric abnormality. Participants with positive *Mycobacterium tuberculosis* culture were excluded from case definition analyses. Statistical analyses were conducted with Stata version 12 (StataCorp, College Station, TX).

## RESULTS

**Figure 1.**
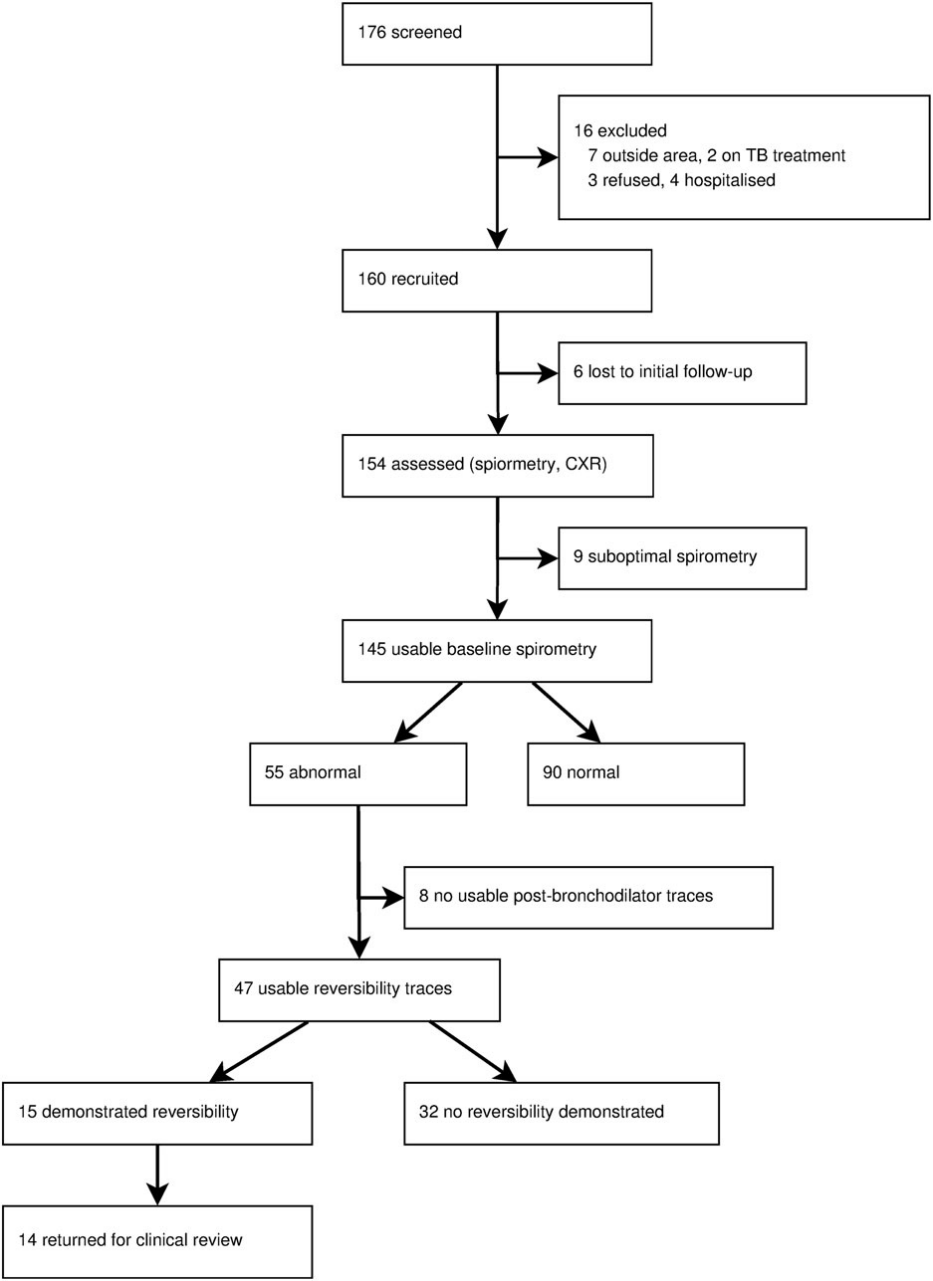
Study flowchart. Flow diagram illustrates participant retention and quality of spirometry throughout the study. CXR, chest x-ray.

The flow and baseline characteristics of the 160 participants are shown in Figure 1 and Table 1, respectively. All children in the study were black African, with a mean age of 11.1 years (SD = 2.1). One hundred fourteen (71.7%) were established on ART, and a further 46 (28.3%) had an established diagnosis of HIV but were not eligible for ART according to national guidelines. Median CD4 counts in these groups were 698 cells/µL and 406 cells/µL, respectively (*P* < .001, Wilcoxon rank-sum test). Perinatal acquisition of HIV was assumed after a systematic review of participants' risk factors: 16 (10.3%) participants had received blood transfusion; 11 (6.9%) participants had previous surgery; 53 (33.1%) participants had injections outside the healthcare setting, including escarification; 3 (1.9%) participants reported sexual abuse; none reported other sexual activity. Eighty-nine (56%) participants had no risk factors other than maternal orphanhood or known HIV infection, although reported factors for other transmission routes were higher than in the Zimbabwe study [13]. Previous respiratory complaints were common: 30 (18.8%) participants had been treated for TB; 20 (12.5%) participants had been treated for asthma; and 13 (8%) participants were treated for chest infection requiring hospitalization. Household air pollution was common. Seventy-six (47.5%) and 117 (73.1%) of households used biomass fuel as the predominant energy source for lighting and cooking, respectively. Passive smoking was reported in 23 (14.5%) participants.

**Table 1.** Demographic and Clinical Characteristics

Characteristic	n (%) unless stated
Age, median years (IQR)	11.1 (9.5–12.4)
Sex, female (%)	80 (50.0%)
Age at HIV diagnosis, median years (IQR)	7.9 (5.8–9.8)
Child aware of HIV diagnosis,^a^ n (%)	65 (41.1)
CD4 count,^c^ median cells/µL (IQR)	572 (370–876)
Taking cotrimoxazole prophylaxis, n (%)	159 (99.4%)
On ART,^a^ n (%)	114 (71.7%)
Duration of ART,^b^ median years (IQR)	3.5 (1.3–4.6)
Chest infection in preceding year, n (%)	30 (18.8%)
Cough, n (%)	60 (37.5)
Sputum produced, n (%)	32 (20.0)
Wheezing in last 12 months, n (%)	13 (8.1)
Breathlessness (NYHA class), n (%)	
0	85 (53.1)
1	9 (5.6)
2	11 (6.9)
3	9 (5.6)
4	46 (28.8)
Stunted [HFA z ≤2], n (%)	89 (55.6%)
WFH z-score, mean (SD)	−0.82 (±1.09)
Finger clubbing, n (%)	34 (22.1)
Resting pulse rate, median min^−1^ (IQR)	87.0 (76.0–98.5)
Resting tachypnoea [>24/min], n (%)	57 (35.6)
Resting hypoxia [SpO_2_ <92%], n (%)	33 (20.6)
Normoxemia but desaturates >4*%, n (%)*	29 (18.1)

Abbreviations: ART, antiretroviral therapy; HFA, height for age; HIV, human immunodeficiency virus; IQR, interquartile range; NYHA, New York Heart Association breathlessness scale; SD, standard deviation; WFH, weight for height.

^a^Continuous data are represented as median (IQR).

^b^Missing data n = 1.

^c^Unknown n = 15; data unavailable n = 3.

It is worth noting that 91 (56.9%) participants had 1 or more of the following symtoms: cough; moderate or severe dyspnoea New York Heart Association ([NYHA] grade III and IV); wheeze. Of those with previous pulmonary TB, 6 (3.8%) had received more than 2 courses. In the preceding year, 34 (21.3%) had used antibiotics for a lower respiratory tract infection: 17 (10.6%) had received multiple courses.

### Quality of Life

Participants reported high quality of life in 6 domains (median scores of 88.9–100.0 for emotional, eating, body image, treatment burden, respiratory, and digestion), where 100 is the maximum score. Social and physical activity domains had the lowest median scores of 57.1 (interquartile range [IQR], 47.6–57.1) and 83.3 (IQR, 55.6–100.0), respectively.

### Developing a Case Definition and Phenotypes of Chronic Lung Disease

An association matrix was used to investigate potential case definitions (Supplementary Table 1). Two patients with active TB were excluded from this analysis. Two candidate phenotypes were postulated: one characterized by cough (37.5%; 95% confidence interval [CI], 30.0%–45.1%) and the other by hypoxia or desaturation at submaximal exercise (38.8%; 95% CI, 31.1–46.4%). Although these might be expected to commonly coexist, only 22 (13.8%; 95% CI, 8.4%–19.1%) participants had both. There was lack of agreement between these 2 variables over that expected by chance alone (expected agreement 52.8%, observed agreement 51.3%, kappa = −3.3%). Therefore, we further characterized those 2 proposed phenotypes (CLD cough and CLD hypoxia) (see Figure 3). Table 2 summarizes univariable and multivariable analysis of risk factors for chronic cough and hypoxia or desaturation individually.

**Table 2.** Risk Factors for Chronic Lung Disease Defined by Presence of Cough and Hypoxia

Characteristic	Presence of Cough				Presence of Hypoxia or Desaturation			
	Univariate OR (95% CI)	*P*	Multivariate^a^ OR (95% CI)	*P*	Univariate OR (95% CI)	*P*	Multivariate^a^ OR (95% CI)	*P*
Distal (Indirect) Factors								
Sex, female	0.72 (0.38–1.38)	.322			1.24 (0.65–2.35)	.514		
Age, years	0.99 (0.84–1.16)	.883	1.00 (0.81–1.23)	.98	0.97 (0.82–1.14)	.68	0.95 (0.79–1.13)	.54
Orphaned (1+ parent died)	0.65 (0.34–1.24)	.192			1.36 (0.71–2.6)	.348		
HFA, z-score	0.85 (0.64–1.12)	.25			0.90 (0.68–1.18)	.451		
WFH, z-score	0.96 (0.71–1.30)	.798			0.91 (0.67–1.22)	.515		
ART prescribed	0.91 (0.45–1.84)	.788	0.79 (0.34–1.83)	.59	0.64 (0.32–1.28)	.207	0.83 (0.35–1.96)	.67
Age at which ART started, years	0.94 (0.82–1.08)	.372			0.92 (0.8–1.06)	.227		
CD4^c^ <100	1.93 (0.53–7.04)	.32			4.03 (0.99–16.39)	.051	3.87 (0.91–16.42)	.066
100–199	1.93 (0.53–7.04)	.32			1.72 (0.47–6.30)	.41		
200–349	1.50 (0.52–4.31)	.45			0.79 (0.26–2.41)	.67		
>349	1.00	–			1.00	–		
Proximal (Direct) Factors								
Previous TB	1.49 (0.69–3.22)	.316			1.51 (0.7–3.24)	.295		
Smoker in household	0.44 (0.15–1.25)	.104			0.49 (0.18–1.33)	.145		
NYHA grade 3 or 4	1.65 (0.84–3.24)	.148			0.77 (0.39–1.52)	.45		
Wheeze	11.47 (2.44–53.83)	<.001	6.94 (1.38–34.95)	.019	0.44 (0.12–1.66)	.198		
CFQ-R physical domain	0.98 (0.97–0.99)	<.001	0.98 (0.97–1.00)	.017	1.00 (0.99–1.01)	.623		
Clubbing	1.19 (0.54–2.63)	.666			2.28 (1.04–4.99)	.038	1.28 (0.50–3.25)	.60
Respiratory rate, >25/min at rest	0.98 (0.50–1.93)	.948			3.00 (1.52–5.92)	.001	2.39 (1.06–5.38)	.032
Pulse rate, beats/min	1.01 (0.99–1.03)	.185			1.00 (0.98–1.01)	.585		
CXR abnormality^b^	3.00 (1.15–7.85)	.023	3.43 (1.01–11.65)	.048	1.04 (0.4–2.7)	.941		
Abnormal spirometry	2.61 (1.29–5.28)	.007	2.09 (0.94–4.66)	.072	1.65 (0.83–3.28)	.153		

Abbreviations: ART, antiretroviral therapy; CI, confidence interval; CFQ-R, Cystic Fibrosis Questionnaire-Revised; CXR, chest x-ray; HFA, height for age; NYHA, New York Heart Association breathlessness scale; OR, odds ratio; TB, tuberculosis; WFH, weight for height.

^a^Adjusted for priori variables: age, sex, and being on ART and significant distal and proximal variables.

^b^Defined here as consolidation, volume loss, or lymphadenopathy because other findings were nondiscriminatory.

^c^Comparator population is those with CD4 >349. Participants diagnosed with pulmonary tuberculosis are not included in this analysis.

Chronic lung disease cough was not associated with any indirect factors, but it was significantly associated with wheeze (odds ratio [OR] = 11.47; 95% CI, 2.44–53.83), abnormal chest radiograph (OR = 3.00; 95% CI, 1.15–7.85), and abnormal spirometry (OR = 2.61; 95% CI, 1.29–5.28). Participants with higher levels of exercise tolerance as measured by CFQ-R physical domain had reduced odds of CLD cough (OR = 0.98; 95% CI, 0.97–0.99). After multivariable analysis, wheeze (OR = 6.94; 95% CI, 1.38–34.95; *P* = .019), CFR-Q physical domain (OR = 0.98; 95% CI, 0.97–1.00; *P* = .017), and abnormal chest radiograph (OR = 3.43; 95% CI, 1.01–7.85; *P* = .048) remained significant.

Chronic lung disease hypoxia had more limited univariate predictors: only finger clubbing (OR = 2.28; 95% CI, 1.04–4.99) and respiratory rate (OR = 3.00; 95% CI, 1.52–5.92). Only resting tachypnoea remained significant in multivariable modeling adjusted for a priori variables. There was weak evidence for the association of CD4 <100 cell/µL and CLD hypoxia (OR = 3.87; 95% CI, 0.99–16.39; *P* = .051) compared with children having a CD4 count of 350 or more.

### Spirometry

**Figure 2.**
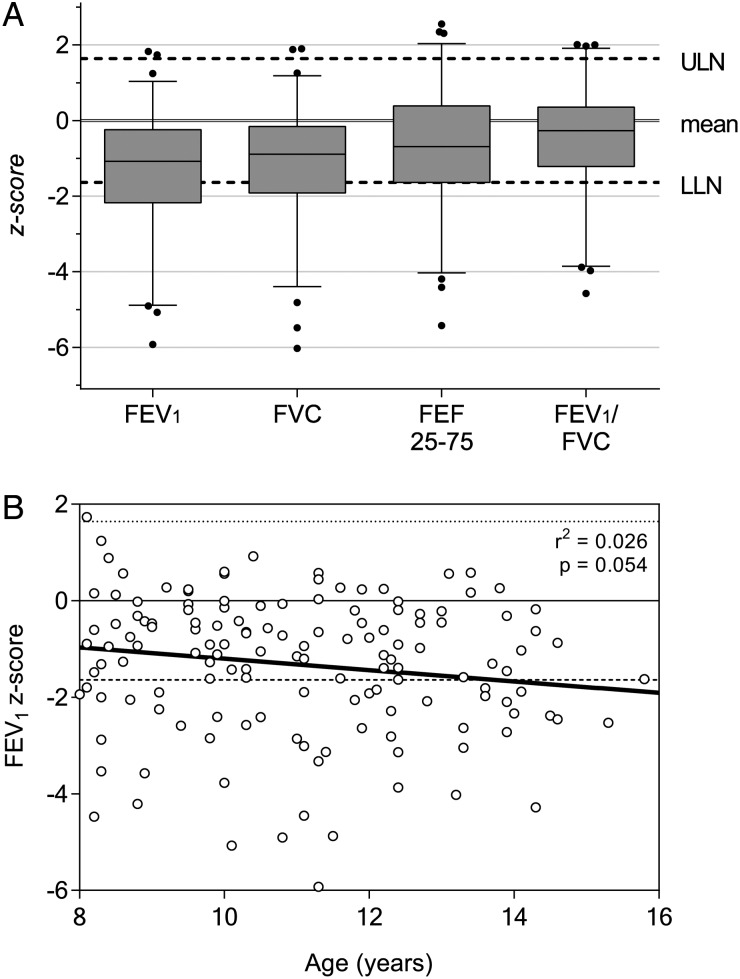
Spirometry results overview. Graphs illustrating the degree and distribution of spirometric abnormality. (A) Boxes represent 25th and 75th percentiles, whiskers represent 10th and 90th percentiles, with outliers shown as individual dots. Upper limit of normal (ULN) and lower limit of normal (LLN) drawn by dashed line at +1.64 standard deviation (SD) and −1.64 SD from the mean, respectively. (B) Forced expiratory volume (FEV_1_) z-score for all participants as a function of age. Linear regression model is shown as a solid line. There is a nonsignificant tendency to reducing FEV_1_ with increasing age in this cohort (*r*^2^ = 0.026; *P* = .054). Similar results for forced vital capacity obtained (results not shown).

Spirometry results for 145 participants are summarized in Table 3 and Figure 2A. Median FEV_1_ and FVC were reduced compared with international reference ranges (1.31 SD and 0.89 SD below expected, respectively). Categorically, 90 (62.1%) participants had normal spirometry, 26 (17.9%) participants had obstructive defects, and 29 (20.0%) participants had reduced FVC. Fewer individuals were classified as having abnormalities using local compared with international reference range (43 vs 55). Within our cohort, FEV_1_ z-score did not significantly decline with age (Figure 2B; *r*^2^ = 0.026; *P* = .054).

**Table 3.** Spirometric **I**ndices*

Baseline Spirometry (n = 145)	GLI Reference [10]	Local Reference
FEV_1_	−1.31 (−2.10 to −0.27)^†^	92.2 (79.5 to 104.6)^‡^
FVC	−0.89 (−1.91 to −0.18)^†^	93.9 (81.8 to 104.2)^‡^
FEV_1_/FVC	−0.27 (−1.21 to 0.35)^†^	87.9 (82.1 to 91.6)^§^
FEF25-75%	−0.69 (−1.63 to 0.38)^†^	Not available
No abnormality, n (%)	90 (62.1%)	102 (70.3%)
Obstruction, n (%)	26 (17.9%)	18 (12.4%)
Reduced FVC, n (%)	29 (20.0%)	25 (17.2%)
Reversibility Testing	FEV % Change	Reversible, n (%)
Reduced FVC pattern (n = 26)	2.6 (−3.6 to 9.5)	8 (30.8%)
Obstructive pattern (n = 21)	3.3 (−4.3 to 12.1)	7 (33.3%)
All (n = 47)	2.7 (−4.3 to 10.1)	15 (31.9%)

Abbreviations: FEF25–75%, forced expiratory flow at 25%–75%; FEV_1_, forced expiratory volume in 1 second; FVC, forced vital capacity; GLI, Global Lung Initiative; IQR, interquartile range.

*Continuous data presented as median (IQR) due to skewed distributions. Includes all traces meeting ATS criteria (grades A and B), n = 145 at baseline, n = 47 for reversibility testing.

^†^Median z-score (IQR).

^‡^Median percentage of predicted (IQR).

^§^Median percentage (IQR).

**Figure 3.**
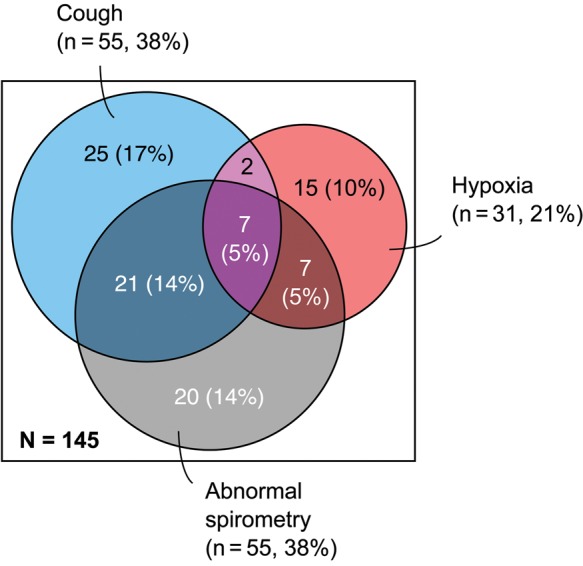
Proposed CLD phenotypes. Proportional areas diagram illustrating the proposed CLD phenotypes, “cough” and “hypoxia”, and their overlap with individuals with abnormal spirometry. Percentages indicate the proportion of the entire study population for which spirometry data were available.

Fifty-five participants had abnormal spirometry, 47 of whom adequately completed postbronchodilator testing. Median change in FEV_1_ was 2.9% (IQR, −4.3 to 9.2). Reversibility threshold of 12% increase was met in 7 (33.3%) of those with obstructive abnormalities and 8 (30.8%) with reduced FVC. When reviewed 4 weeks afterwards, despite being given salbutamol, only 2 participants continued to use their inhaler, and none reported symptomatic improvement.

### Chest Radiograph Abnormalities

The majority of radiographs had at least 1 abnormality (n = 110, 68.8%). Upper or lower zone preponderance were uncommon (n = 12, 10.9% and n = 13, 11.8%, respectively) compared with mid-zone abnormality. The most frequent abnormality was ring or tramlining pattern (n = 90, 56.3%). Two abnormalities (airspace shadowing and volume loss) were discriminatory for both CLD phenotypes (Table 2) and spirometric abnormality (Supplementary Table 3). Air space shadowing (n = 10, 6.3%) and loss of volume (n = 3, 1.9%) were associated with reduced FEV_1_ (*P* = .0032 and *P* = .010, respectively). Other radiographic findings were not significantly associated with differences in FEV_1_ or FVC z-score.

### Clinical Associations of Lung Function

Potential associates of abnormal spirometry were investigated (Supplementary Data, Table 2). Only 1 strong association emerged: individuals reporting cough for more than 1 month were 2.9 times more likely to have abnormal spirometry (95% CI, 1.21–7.10).

### Microbiological Findings

Sputum was obtained from 32 of 60 participants with cough, with the remainder unable to expectorate. There were 6 positive mycobacterial cultures; 2 *M tuberculosis* and 4 nontuberculous mycobacteria.

## DISCUSSION

This study demonstrates a high burden of symptoms in children aged 8 to 16 with vertically acquired HIV, consistent with a similar study from Zimbabwe [14]. Over half of our participants were coughing, wheezy, or breathless. Within our cohort, there are 2 definable, common, and independent phenotypes: children who cough (CLD cough), and those who have hypoxia at rest or desaturate with submaximal exercise (CLD hypoxia). Neither phenotype was associated with antiretroviral treatment.

For the CLD-cough phenotype, cough, wheeze, and functional breathlessness were commonly associated with each other and also with radiological abnormalities of airspace shadowing and volume loss where parenchymal lung disease was likely. Previous treatment for TB was not a significant risk factor for this phenotype, and symptoms were mostly chronic (individuals with symptoms of acute infection were excluded). Abnormal spirometry was associated with this phenotype, but there was no preponderance of obstructive or restrictive types.

The CLD-hypoxia phenotype was predictably associated with tachypnoea. There was a suggestion that very low CD4 counts (<100) predicted hypoxia. Low numbers of individuals in this group limited our power to detect a difference. Although not independently associated, there was a higher than expected rate of finger clubbing in these individuals.

Chronic lung disease in these children is likely to be multifactorial and therefore difficult to clearly define [15]. Frequent bacterial, mycobacterial, and viral respiratory infections were reported in this population (18.8% of our cohort had received treatment for chest infection in the preceding year), and these can also contribute to bronchiectasis. Consistent with underlying bronchiectasis, there was a high rate of finger clubbing, reduced lung function, and radiological abnormalities consistent with bronchiectasis. These features are insensitive and nonspecific for its diagnosis in isolation [16]. A direct effect of HIV and chronic inflammation of the airways might cause reduced lung capacities and chronic chest x-ray findings, including lymphadenopathy. Findings that the pulmonary microbiota can be altered in adult HIV, notably for *Tropheryma whipplei* bacteria [17], raise the possibility that these changes may reflect or drive long-term disease in the airways, including chronic inflammation. High-resolution computerized tomography findings from similar patients in Zimbabwe that are suggestive of airways disease [18] can represent the final common pathway of many diseases, including postinfective change, although this is uncommon outside of allogeneic transplantation [19]. In our study, the clinical syndrome of CLD cough including nonreversible spirometry findings would be consistent with such pathology [20].

Toro et al [21] demonstrated a high burden of pulmonary lymphoid hyperplasia and lymphoid interstitial pneumonitis (LIP) in early life associated with a wide variety of radiological changes, of which reticular infiltrates are most typical [22]. Early Western cohorts including the P2C2 study noted high rates of LIP and reported chest radiograph with LIP suggestive changes [23], but this condition has been almost eliminated with effective ART provision [24]. Our participants were considerably older than the usual age of LIP presentation [25], and they started ART later. In this case, the 2013 WHO guidelines to start all HIV- positive children under 5 years on ART may improve rates of CLD in the future.

A Zimbabwean study has shown a high frequency of cardiac abnormalities and cor pulmonale in adolescents with vertically transmitted HIV infection [14]. This raises the possibility that CLD hypoxia might represent pulmonary vascular disease or interstitial lung disease with secondary cardiac involvement.

The degree of impairment of lung function is marked when measured against both internationally used and locally derived reference ranges. Adult HIV patients in the United States have higher rates of asthma than the general population [26], but the generalizability to our age group and geography is uncertain. The International Study of Asthma and Allergies in Childhood (ISAAC) study [27] did not cover Malawi, but prevalence of wheeze was 15.9% in English-speaking African countries. In our study, bronchodilator reversibility was minimal, and rates of wheeze were similar to regional rates in the general population, suggesting that asthma was unlikely to be a predominant pathology. The FEV_1_ improvement after inhaled bronchodilator was disappointing, and on average it was indistinguishable from zero. Therefore, it is possible that even those with >12% increase in FEV_1_ may represent bias related to regression to the mean. No participants found salbutamol helpful at 4 weeks: effective treatment options are urgently needed.

Within our cohort, there is no strong evidence for clinically significant decline in lung function with age. However, this could be confounded by age of ART initiation, and a longitudinal study to specifically examine this is in progress. Declining FEV_1_ is reported in CLDs such as cystic fibrosis and chronic obstructive pulmonary disease. In other cohorts (chronic coughers with bronchiectasis which presented in childhood), FEV_1_ declined with age, but this was apparent only after many years [28]. Some decline may be artefactual relating to growth and maturation delay, although the significant baseline abnormality suggests that earlier life events have already strongly affected the lung architecture. In any case, the lack of clinical predictors of lung function abnormality suggests that considerable lung abnormalities, through intercurrent disease or other effects on lung growth, may not be identified unless spirometry is performed. Rates of reported household biomass fuel use were typical for many sub-Saharan countries. This important public health problem may have contributed to reduced lung function in our population [29].

Our data are limited by (1) the cross-sectional nature of the study, (2) the lack of total lung volume and transfer factor measurements, and (3) the absence of noninfected controls. Reversibility studies might be more easily interpreted with either universal reversibility testing or a control arm, but this was not possible within our study. We did not have access to HRCT imaging, echocardiography, or post mortem tissue biopsies, which would define the pathologies more clearly, and TB screening was limited by suboptimal diagnostics.

Prospective studies should examine our definition of the 2 phenotypes in relation to pathophysiology in a cohort in which intensive investigation is possible, for example, with high-resolution computed tomography scanning, echocardiography, and, possibly, autopsy studies. If the phenotypes correlate with disease (we hypothesize cough with bronchiectasis or bronchiolitis obliterans and hypoxia with interstitial lung disease), this could be useful to clinicians where such investigations are not available. Longitudinal cohort studies should assess long-term change in symptoms and lung function in CLD, and they would facilitate therapeutic trials of immunomodulation (for example, prednisolone in obliterative bronchiolitis) or antimicrobials (azithromycin in bronchiectasis).

## CONCLUSIONS

Widespread evidence of pulmonary disease presented here adds to the case for treatment of all HIV-infected children with antiretrovirals irrespective of CD4 count. At the very least, as a WHO HIV Stage 3 criterion, there should be a strong emphasis on identifying children with CLD and establishing early ART in those individuals. For this purpose, simple clinical definitions of CLD cough (in the absence of TB) and CLD hypoxia could be useful to clinicians in healthcare settings with few resources.

## Supplementary Data

Supplementary materials are available at the Journal of The Pediatric Infectious Diseases Society online (http://jpids.oxfordjournals.org).
